# Thermoelectric Energy Harvesting from Single-Walled Carbon Nanotube Alkali-Activated Nanocomposites Produced from Industrial Waste Materials

**DOI:** 10.3390/nano11051095

**Published:** 2021-04-23

**Authors:** Maliheh Davoodabadi, Ioanna Vareli, Marco Liebscher, Lazaros Tzounis, Massimo Sgarzi, Alkiviadis S. Paipetis, Jian Yang, Gianaurelio Cuniberti, Viktor Mechtcherine

**Affiliations:** 1 Institute of Construction Materials, Faculty of Civil Engineering, Dresden University of Technology, 01069 Dresden, Germany; maliheh.davoodabadi@tu-dresden.de (M.D.); viktor.mechtcherine@tu-dresden.de (V.M.); 2 Institute for Materials Science and Max Bergmann Centre of Biomaterials, Dresden University of Technology, 01069 Dresden, Germany; massimo.sgarzi@tu-dresden.de (M.S.); gianaurelio.cuniberti@tu-dresden.de (G.C.); 3 Dresden Center for Nanoanalysis (DCN), Dresden University of Technology, 01069 Dresden, Germany; 4 Department of Civil Engineering, School of Naval Architecture, Ocean and Civil Engineering, Shanghai Jiao Tong University, Shanghai 200240, China; j.yang.1@sjtu.edu.cn; 5 Department of Materials Science and Engineering, University of Ioannina, 45110 Ioannina, Greece; i.vareli@uoi.gr (I.V.); paipetis@uoi.gr (A.S.P.); 6 Center for Advancing Electronics Dresden (CfAED), Dresden University of Technology, 01069 Dresden, Germany; 7 Dresden Center for Computational Materials Science (DCMS), Dresden University of Technology, 01069 Dresden, Germany

**Keywords:** alkali-activated nanocomposites, single-walled carbon nanotubes, thermal energy harvesting, thermoelectric generator, multifunctional waste materials, green construction

## Abstract

A waste-originated one-part alkali-activated nanocomposite is introduced herein as a novel thermoelectric material. For this purpose, single-walled carbon nanotubes (SWCNTs) were utilized as nanoinclusions to create an electrically conductive network within the investigated alkali-activated construction material. Thermoelectric and microstructure characteristics of SWCNT-alkali-activated nanocomposites were assessed after 28 days. Nanocomposites with 1.0 wt.% SWCNTs exhibited a multifunctional behavior, a combination of structural load-bearing, electrical conductivity, and thermoelectric response. These nanocomposites (1.0 wt.%) achieved the highest thermoelectric performance in terms of power factor (PF), compared to the lower SWCNTs’ incorporations, namely 0.1 and 0.5 wt.%. The measured electrical conductivity (*σ*) and Seebeck coefficient (*S*) were 1660 S·m^−1^ and 15.8 µV·K^−1^, respectively, which led to a power factor of 0.414 μW·m^−1^·K^−2^. Consequently, they have been utilized as the building block of a thermoelectric generator (TEG) device, which demonstrated a maximum power output (*P_out_*) of 0.695 µW, with a power density (PD) of 372 nW·m^−2^, upon exposure to a temperature gradient of 60 K. The presented SWCNT-alkali-activated nanocomposites could establish the pathway towards waste thermal energy harvesting and future sustainable civil engineering structures.

## 1. Introduction

Alkali-activated materials can be considered as low-carbon and energy-efficient construction materials developed by geopolymer technology [1,2,3]. These binders are produced by the geopolymerization of precursors reacting with alkaline activators. The alkali-activated amorphous gel is composed of the geopolymeric and cementitious phases, in their coexistence or hybrid forms [1,2,4,5,6]. Precursors are multi-oxide components, which can be supplied from industrial wastes streams or byproducts [1]. The most favorable industrial waste-originated precursors are fly ash and slag, but agricultural wastes such as corn cob ash and palm oil fuel ash are proposed for total or partial substitution, because of being byproducts of biomass factories, and thus, greener [1,2,7]. The second main element of alkali-activated materials is the alkaline activator, made of mostly alkali silicates or alkali hydroxides [8]. Alkaline activators can be introduced into the mix in solution and solid state to produce two-part and one-part alkali-activated materials, respectively. In the one-part approach, water should be included in the mix design [9,10]. Replacing factory-synthesized activators with recycled waste-based substances will be a more sustainable methodology [11,12].

Apart from the conventional applications, such as being a mechanical or chemical durable binder [13,14], alkali-activated materials exhibit interesting multifunctional characteristics [15,16]. Existence of an ion-rich framework and consequently a very high ionic mobility in the presence of pore water can be an evidence of alkali-activated materials’ inherent sensing potential [17,18,19]. The ionic and electrolytic conductivities of alkali-activated materials have been reported in the ranges of 1.5–1.7 × 10^−2^ S·m^−1^ [20], 1.4–5.2 × 10^−2^ S·m^−1^ and 3.7 × 10^−1^–0.15 × 10^1^ S·m^−1^ [21], 0.8–11 × 10^−2^ S·m^−1^ [22], and 8 × 10^−6^ S·m^−1^ [23]. In parallel, there is a tremendous effort to establish electronic conductivity within the alkali-activated microstructures by adding conductive carbon-based nanofillers, notably carbon nanotubes [16,17,24,25,26,27]. A conductivity of 1.9 × 10^−3^ S·m^−1^ was measured for 0.2 wt.% SWCNT-incorporated potassium-clay geopolymer, while the reference had a conductivity of 9.8 × 10^−4^ S·m^−1^ [28]. Slag alkali-activated nanocomposites (2.5 wt.% of CNTs) reached a conductivity of 45 × 10^−3^ S·m^−1^ compared to its reference with the conductivity of 15 × 10^−3^ S·m^−1^ [29]. Adding 1 wt.% MWCNT into fly ash geopolymer composites could increase the conductivity from 1.5 S·m^−1^ for reference to 3 S·m^−1^ for nanocomposite [30].

As multifunctional and smart materials, alkali-activated materials are expected to be utilized in novel applications toward future visions. One of these upcoming applications is energy harvesting. Considering the massive use of construction materials in urban and industrial areas, waste heat produced in these environments can be captured and converted into electrical energy by using thermoelectric materials and generators [31,32]. According to the literature, cementitious composites can present thermoelectric properties after modification by carbon or metallic-oxide nanoparticles [33,34]. However, utilization of waste-originated materials, i.e., alkali-activated materials and geopolymers, will be more sustainable and eco-friendlier for thermoelectric energy harvesting [35,36].

The application of a temperature gradient to the end terminals of a thermoelectric material can produce electrical potential. The Seebeck coefficient can be obtained from the negative ratio of these gradients (voltage to temperature) that will be negative for electron carriers or n-type materials, and positive for hole carriers or p-type materials. The product of the Seebeck coefficient squared and electrical conductivity will yield the power factor [31,32,37]. For a large-scale development, thermoelectric blocks, i.e., thermoelements, can be appropriately interconnected to constitute a generator device. These solid-state thermoelectric devices, consisting of multiple discrete thermoelements, will be able to directly convert waste heat energy to electricity [38,39].

So far, thermoelectric properties of alkali-activated materials have been investigated among some limited cases without inclusion of any additive. Seebeck coefficients of metakaolin and fly ash geopolymers activated by a combination of Na_2_SiO_3_ and 12 M KOH cured at 50 °C were −13.28 and −3.36 µV·C^−1^, respectively. These values were −10.73 and −1.63 µV·C^−1^ with 4 M KOH [35]. Despite that, in other research, the documented Seebeck coefficient of metakaolin-geopolymers activated by a mixture of Na_2_SiO_3_ and NaOH was unexpectedly high, 1736–2170 µV·C^−1^. The three-dimensional aluminosilicate nanostructure of that assessed geopolymer was expressed as the main reason behind this extraordinarily high Seebeck effect, similar to what takes place in metal oxide thermoelectric material [36]. Nevertheless, it seems that geopolymeric ionic mobility and diffusion can cause such exceptionally large Seebeck effects, which are not steady over time. Apart from in the above-mentioned pieces of research, the authors of this research could not find any reported alkali-activated thermoelectric generator in the accessible scientific literature.

By those arguments and grounds, the present study will be among the first attempts at assessing the thermoelectricity of alkali-activated SWCNT nanocomposites and fabricating/demonstrating for the first time an alkali-activated SWCNT-based thermoelectric generator device, allowing for large-scale thermal energy harvesting and practical applications. A blend of fly ash and slag (GGBS), activated by a powder sodium silicate, is the main component of the investigated one-part alkali-activated composites. A TEG device consisting of ten (10) p-type serially interconnected thermoelements has been fabricated and tested for the first time, which produced a voltage output of 7.4 mV with a maximum power (*P_max_*) of 0.695 µW upon exposure to a through-thickness thermal difference of ∆*T* = 60 K. The total voltage and power output values could be further enhanced towards large-scale thermal energy harvesting by the fabrication of TEG devices with a higher amount of p-type or alternating p-n serially interconnected thermoelements.

## 2. Materials and Methods

### 2.1. Materials

TUBALL^TM^ graphene nanotubes (OCSIAL Europe, Leudelange, Luxembourg) were used as conductive nanomaterials in this research. They are commonly known as single-walled carbon nanotubes (SWCNTs), (Table 1). SWCNTs were dispersed in the deionized water by sodium dodecylbenzenesulfonate (SDBS) as surfactant. SDBS was technical grade with the linear formula of CH_3_(CH_2_)_11_C_6_H_4_SO_3_Na and molecular weight of 348.48, according to the supplier data (Merck KGaA, Darmstadt, Germany).

The precursors used to produce one-part alkali-activated composites were fly ash (Steament^®^ H-4 FA) supplied from Steag Power Minerals GmbH, Dinslaken, Germany, and ground granulated blast-furnace slag (GGBS) from Opterra GmbH, Leipzig, Germany (Table 2). The activation was carried out by sodium disilicate powder (Wöllner GmbH, Ludwigshafen, Germany), commercially known as Sikalon^®^ A, with a molar ratio (SiO_2_/Na_2_O) of approximately 2.1 and water content of around 16%, according to the factory descriptions. The chemical composition of precursors in weight percentage (wt.%) was obtained by EDX analysis and it has been discussed in detail in our previous work [13].

### 2.2. Methods

#### 2.2.1. Alkali-Activated Nanocomposites’ Fabrication

Alkali-activated nanocomposites were prepared with the same formula but containing different contents of SWCNT according to the Table 2. SWCNTs were incorporated based on 0.1, 0.5, and 1.0 of the precursors’ mass percentage. SDBS was diluted to the concentration of 10% (in mass) in deionized water and added with the mass ratio of 1 (SDBS to SWCNT). The amounts of precursors and powder activator were kept identical in all nanocomposites, and water-to-solid ratio was considered 0.45 to prepare adequate media for SWCNT dispersion.

**Figure 1:**
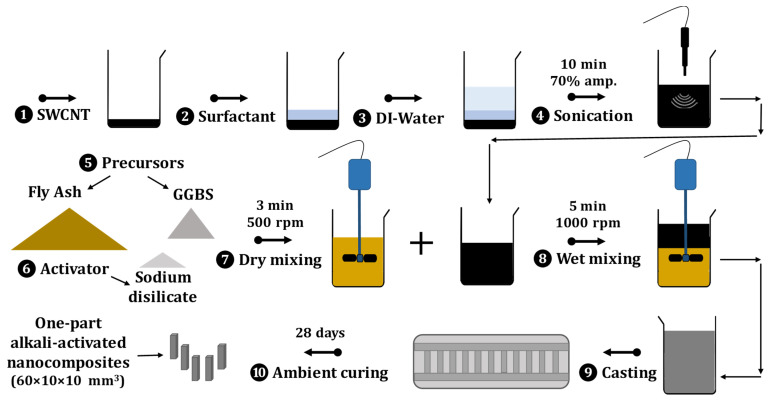
Schematic view of one-part SWCNT-alkali-activated nanocomposites’ synthesis in chemical laboratory scale.

The general fabrication procedure for one-part SWCNT-alkali-activated nanocomposites is illustrated in Figure 1. First, the mixture of SWCNT, SDBS, and deionized water was ultrasonicated for 10 min with a Sonopuls HD 2200 ultrasonic homogenizer (Bandelin Electronic GmbH & Co. KG, Berlin, Germany), equipped with an MS 73 probe. Ultrasonication was applied in cycles of 50% and 70% amplitude for 10 min. Subsequently, all powders (fly ash, GGBS, and sodium disilicate) were mixed by the overhead stirrer HT-120DX (Witeg labortechnik GmbH, Wertheim, Germany) for 3 min at 500 rpm. The sonicated nanosolution was gradually added to the solids blend and mixed for 5 more minutes at 1000 rpm. For shear-mixing purpose, a TR 20 radial-flow impeller 28 mm (Heidolph Instruments GmbH & CO. KG, Schwabach, Germany) was attached to the overhead stirrer. After shear-mixing, the obtained slurry was cast into a plastic mold and covered with a plastic bag for 24 h. Lastly, the specimens were demolded and cured in the ambient environment of the chemical laboratory for 28 days. The final rectangular prismatic nanocomposites were 60 mm long with a cross-section of 10 × 10 mm^2^. Experimental details and elaboration of methodologies were optimized in the previous research of the authors [13].

#### 2.2.2. Electrical and Thermoelectric Properties Characterization

A typical 4-probe technique using a commercial four-point probe system (Ossila Ltd., Sheffield, UK) was employed for the electrical sheet resistance (*R_s_*) measurements. The electrical conductivity (*σ*) of the nanocomposites was calculated according to [40]:

(1) $$\sigma =\left(\frac{1}{R_{s}}\right)\cdot \left(\frac{L}{A}\right)\cdot \left(\frac{\ln2}{\pi }\right),$$

where *L* is the length and *A* is the cross-section of each sample. For measuring the *R_s_*, silver paste was applied on the contact surface of the nanocomposites and probes to eliminate the contact resistance and increase the measurements’ accuracy. Reported mean values were derived from six measurements of individual specimens (10 × 10 × 30 mm^3^).

The Seebeck coefficient (*S*) of SWCNT-alkali-activated nanocomposites (10 × 10 × 30 mm^3^) was determined via a laboratory-developed apparatus. To enable the generation of the different temperature gradients (∆*T*), each sample was mounted on two metal blocks. For every measurement, one block was kept at room temperature (*T_cold_*, at 25 °C), while the other (*T_hot_*) was heated up using calibrated temperature-controlled resistors, generating the required ∆*T* of 15 K, 30 K, and 60 K, respectively. Silver wires were wrapped around the two end-point perimeter of every sample in order to derive the thermoelectric voltage measurements. Silver paste was also applied between the sample surface and the wires to minimize any conceivable contact resistance. These silver wires were further connected to an Agilent 34401A 6½ digital multimeter to measure the generated thermovoltage (Δ*V*). In addition, an IR-thermometer (OMEGA OS-VIR50 Dual Laser Video IR Video Thermometer) was engaged to measure the temperature of the two blocks and determine Δ*T*. The Seebeck coefficient was afterwards derived from Δ*V*/Δ*T*. This Seebeck coefficient minus the absolute thermoelectric power of silver (2.34 μV/K) is the absolute thermoelectric power of the sample [41].

#### 2.2.3. Thermoelectric Generator Characterization

The thermoelectric generator (TEG) was fabricated out of 10 SWCNT-alkali-activated thermoelements with a squared shape (10 × 10 × 10 mm^3^). Elements were connected electrically in series and thermally in parallel. Silver conductive tape was applied for connecting the cold side of each element to the hot side of the adjacent one. The open circuit voltage (*V_OC_*), also known as voltage of the TEG (*V_TEG_*), the internal resistance (*R_TEG_*) of the device, and the short-circuit current (*I_sc_*) were measured with an Agilent 34401A 6½ digital multimeter. The TEG device was placed on a hot plate with the hot surface of the device being exposed to 40, 55, and 85 °C, and the cold surface being exposed to the environment (25 °C). As the result, “through-thickness” temperature gradients of ∆*T* = 15 K, 30 K, and 60 Κ were generated, respectively. Finally, a laboratory-developed apparatus was used to conduct the TEG device power output measurements.

#### 2.2.4. Mechanical and Microstructural Properties Characterization

A Zwick 1445 universal testing system (ZwickRoell GmbH & Co. KG, Ulm, Germany) was used for strength measurements of the SWCNT-alkali-activated nanocomposites after 28 days. Bending and compression tests were performed at force capacities of 1 and 10 kN, respectively, and 1 mm·min^−1^ rate. Afterwards, the samples were preserved in isopropanol for 24 h to stop further possible reactions. After evaporation of isopropanol in the ambient conditions (24 h) of the chemical laboratory, the ruptured surfaces of nanocomposites were characterized by a FEI Quanta™ 250 FEG-ESEM, FEI Europe B.V., Eindhoven, The Netherlands.

## 3. Results and Discussion

### 3.1. Thermoelectric Properties

**Figure 2:**
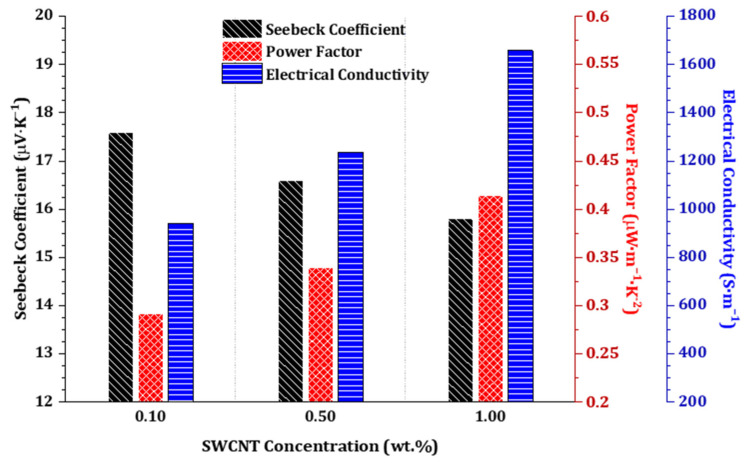
Thermoelectric properties of 28-day one-part SWCNT-alkali-activated nanocomposites.

The electrical conductivity (*σ*), absolute Seebeck coefficient (*S*), and the calculated power factor (*PF*) for the 28-day one-part SWCNT-alkali-activated nanocomposites are presented in Table 3 and Figure 2. The highest conductivity and power factor were obtained for 1.0 wt.% SWCNT nanocomposites (1.66 × 10^3^ S·m^−1^, and 0.414 μW·m^−1^·K^−2^, respectively) while the highest Seebeck coefficient was for 0.1 wt.% SWCNT nanocomposites (17.6 μV·K^−1^). The electrical conductivity of these nanocomposites was extraordinarily high compared to the alkali-activated and geopolymeric counterparts discussed in the introduction. Such negligible resistivity within the nanocomposites might be due to the higher proportion of metallic carbon nanotubes rather than semiconductor ones in the composition of utilized SWCNTs. It is notable that the electrical conductivity of the nanocomposites was double-checked after thermal drying to suppress any ionic or electrolytic conductivity of pore solutions. Since no differences could be observed, it can be concluded that this very high electrical conductivity stems from the percolated SWCNTs’ network. Moreover, the positive values of the Seebeck coefficient suggest a p-type semiconducting behavior, which is a common phenomenon in pristine carbon nanotubes, which exhibit a slightly p-type semiconductor behavior [33,42].

### 3.2. Power Output of the Thermoelectric Generator

**Figure 3:**
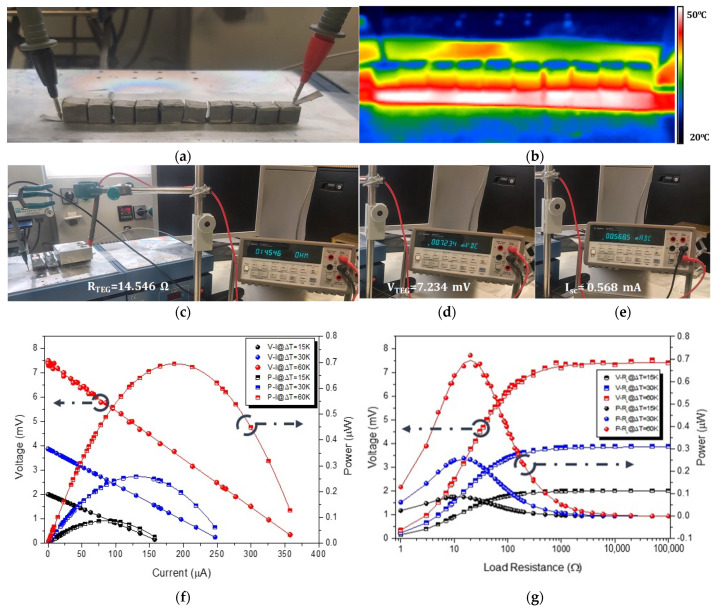
(**a**) The real SWCNT-based TEG device upon being exposed to ∆*T* = 30 K and (**b**) the corresponding IR-T image showing thermal distribution; (**c**–**e**) the digital photos of the TEG device under test at ∆*T* = 60 K (max. tested temperature gradient), showing the device electrical characteristics, i.e., *R_TEG_*, *V_oc_* (equal to *V_TEG_*), *I_sc_*.; (**f**) the experimental output voltage-current (*V*-*I*) and output power-current (*P*-*I*) curves; and (**g**) the *V*-*R_load_* and *P*-*R_load_* curves (**f**,**g**) plots correspond to the measurements upon the device which was exposed to three different Δ*T* values, i.e., 15 K, 30 K, and 60 K, applying different external load resistances (*R_load_*). The peak power at ∆*T* = 15 Κ, ∆*T* = 30 Κ, and ∆*T* = 60 Κ occurred when the *R_load_* matched the internal TEG device resistance (*R_TEG_*).

Figure 3a depicts the SWCNT-alkali-activated-based TEG with the corresponding dimensions, fabricated from the p-type nanocomposites with an achieved power factor of 0.414 μW·m^−1^·K^−2^ (Figure 2). It can be observed that the device consisted of ten (10) thermoelements serially interconnected with silver conductive tape used as the metallic junction for the interconnection of the thermoelements. An infrared thermography (IR-T) image of the TEG device is illustrated in Figure 3b upon being exposed to a thermal gradient of ∆*T* = 30 K.

It is worth mentioning that the expected and theoretical *V_TEG_* would be 4.74 mV taking into consideration the following equation [43]:

*V_TEG_* = *N* × *S* × ∆*T* (2)

where *N* is the number of thermoelements, *S* is the Seebeck coefficient, and ∆*T* is the temperature difference. However, the experimentally measured *V_TEG_* (otherwise defined also as the device *V_out_*) value was 3.85 mV at ∆*T* = 30 K, revealing an individual element contribution of 0.385 mV to the total *V_TEG_* ($\sum_{1}^{10}V_{i}$). This could be reasonably attributed possibly to some electrical contact loss between the electrodes and the thermoelements of the device, or some electrical and/or thermal contact resistance of the TEG device in operation.

The measured, slightly decreased *V_out_* of the TEG device, in comparison to the theoretical and expected one, had furthermore an effect on the maximum electrical power output (*P_max_*) of the SWCNT alkali-activated-based TEG device, which can be derived by the following equation [33]:

(3) $$P_{max\left(theoretical\right)}=\frac{{\left(N\;S\;∆T\right)}^{2}}{4R_{in}}or\;\;\;P_{max\left(experimental\right)}=\frac{∆V_{TEG}^{2}}{4R_{in}}\;\;,$$

where *P_max_* is the maximum output of electrical power and *R_in_* is the internal electrical resistance of the TE generator (or *R_TEG_*).

The expected *P_max_* considering the experimentally measured Seebeck coefficient at thermoelement level (*S* = +15.8 µV·K^−1^) for the SWCNT at 1.0 wt.%, shown in Figure 2, would be 0.789 μW at ∆*T* = 60 K, which was also the highest temperature gradient used in this study to characterize the performance of the TEG device. However, the *P_max_* of the device, considering the experimentally measured *V_out_* and the second part of Equation (3), was calculated to be 0.695 µW at ∆*T* = 60 K. Moreover, it should be mentioned that in the present study, thermoelectrically generated electronic charge carriers were the ones to contribute to the thermoelectric measured effect, in contrast to plausible mixed thermoelectrically generated carriers, i.e., electronic and ionic charge carriers, as reported in other studies, which resulted also in significantly high experimentally measured Seebeck coefficient values [44].

Practical use of the TEG devices requires power generation, which has been measured in this study using a homemade apparatus. Specifically, power measurements on the alkali-activated TEG device were performed for several different load resistances (*R_Load_*), at the three different values of ∆*T* to which the device was exposed. Figure 3f shows the experimental output voltage-current (*V*-*I*) and output power-current (*P*-*I*) curves, while Figure 3g displays the *V*-*R_Load_* and *P*-*R_Load_* curves for each Δ*T*, applying different external load resistances. The continuous lines in all curves have been derived from calculations of the following formula (Equation (4)), giving the output power (*P*):

(4) $$P=I^{2}R_{Load}={\left(\frac{V_{oc}}{R_{in}+R_{Load}}\right)}^{2}R_{Load},$$

where *I* is the output current (defined also as *I_load_*: current which passes through the load), *R_load_* is the load resistance, *R_in_* is the internal resistance of the device (or *R_TEG_*), and *V_oc_* is the open circuit voltage (equal to the measured *V_TEG_*) when the *R_load_* approaches infinity. It can be observed that the output voltage for the different *R_load_* applied was inversely proportional to the output current. 

Moreover, the maximum power generation of 0.695 µW at ∆*T* = 60 K, 0.251 µW at ∆*T* = 30 K, and 0.085 µW at ∆*T* = 15 K (Figure 3), respectively, could be observed when *R_load_* matched with the internal TEG resistance of 14.5 Ω. The *P_max_*_,_ differently known as the maximum electric power dissipated in the load resistor, is accomplished when the load resistance equals the TEG device resistance (*R_TEG_*). Moreover, at 14.5 Ω as load resistance, the thermoelectric power voltage *V_TEG_* was 7.2 mV, compared to the theoretical open circuit voltage (*V_oc_*) of the device; as previously also defined, *V_TEG_* = 7.5 mV at Δ*T* = 60 K, while experimentally measured and theoretical *V_TEG_* values were almost identical at Δ*T* = 15 K (2.1 mV). Above 14.5 Ω, the output voltage (defined also as thermoelectric power voltage: (*V_TEG_*)) continued to increase as it reached the open circuit voltage (*V_TEG_*), but *P* decreased as the load resistance increased. The squared behavior of *V_TEP_* was expected due to the relation $P=V_{load}^{2}/R_{load}$.

Finally, the power density of the TEG device can be determined by the following equation [43].

(5) $$P_{density}=\frac{P_{max}}{N\cdot A}=\frac{{\left(N\cdot S\cdot ∆T\right)}^{2}/\left(4\cdot N\cdot \frac{l}{\sigma \cdot w\cdot d}\right)}{N\cdot w\cdot d}=\frac{S^{2}\cdot \sigma }{4l}\cdot \Delta T^{2},$$

where *w*, *d*, and *l* are the width, thickness, and length of the thermoelements, respectively. The achieved maximum power density of the alkali-activated TEG device was 372 nW/m^2^ at Δ*T* = 60 K, which can be further optimized for future energy harvesting applications.

The maximum electrical power output, power density, and thermoelectric power voltage values of the SWCNT-alkali-activated TEG device, corresponding to different temperature gradients, are summarised in Table 4.

### 3.3. Stability of the Thermoelectric Generator and Performance over Time

**Figure 4:**
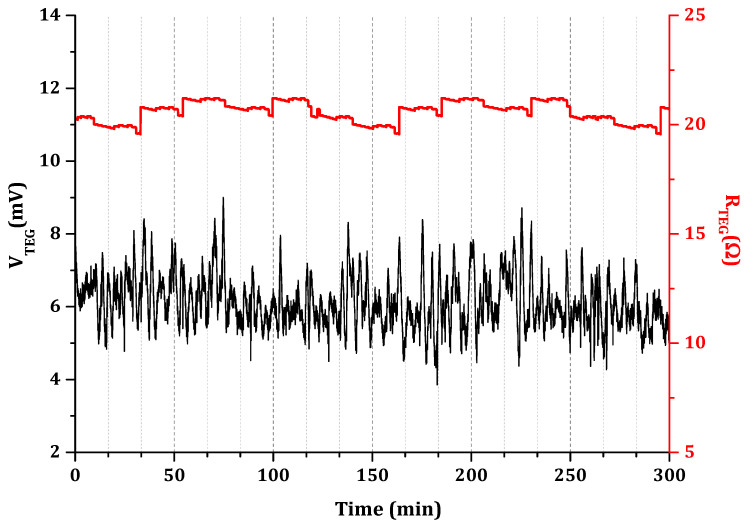
Stability verification of 28-day one-part SWCNT-alkali-activated nanocomposites at Δ*T* = 60 K.

The thermoelectric SWCNT-alkali-activated generator was tested for its stability over time upon being exposed to a temperature gradient of ∆*T* = 60 K. After 5 h of continuous current passing through the generator, a quasi-constant open circuit voltage was recorded. Both the *V_TEG_* and *R_TEG_* of the device were monitored and manifested exceptional stability, as illustrated in Figure 4. This indicates no sign of degradation, e.g., no contact losses due to the continuous ∆*T* exposure for a prolonged period of time.

### 3.4. Mechanical Properties

**Figure 5:**
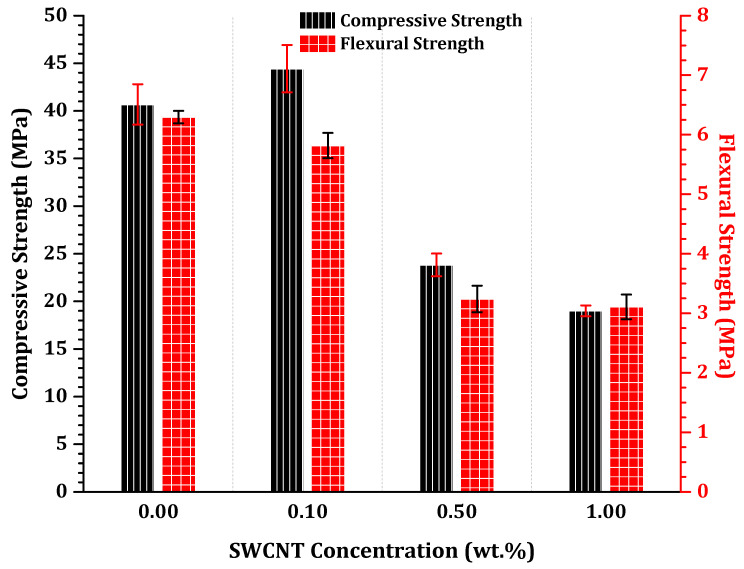
Compressive and flexural strengths of 28-day one-part SWCNT-alkali-activated nanocomposites.

The compressive and flexural strengths of one-part SWCNT-alkali-activated nanocomposites were approximately in the range of 45–20 MPa and 6–3 MPa, respectively, according to the incorporated SWCNT concentrations (Figure 5). The highest compressive and flexural strengths were obtained for 0.1 wt.% nanocomposites; 0.5 and 1.0 wt.% SWCNT nanocomposites could reach almost half the maximum values. This can be traced back to the difficulties in dispersion of SWCNTs in higher contents. With addition of 0.1 wt.% SWCNT, a proper dispersion could be achieved, which enabled sufficient thermoelectric properties besides an adequate mechanical performance. However, 0.5 wt.% SWCNT cannot be completely dispersed. The remaining CNT agglomerates may have acted as defects throughout the nanocomposites, resulting in the observed drop of flexural and compressive strengths. Moreover, in this research SDBS was utilized as the surfactant to disperse SWCNTs, as frequently reported in literature [45,46,47]. Nevertheless, the application of SDBS is also related to foaming during ultrasonication and shear mixing. Since the SDBS-to-CNT ratio was kept constant, the surfactant content rose accordingly with the increase of SWCNT loadings. The entrapped air yielded a porous and non-compact matrix after the hardening of the nanocomposites. The problem herein is especially critical because no de-foaming agent, e.g., tributyl phosphate, was added during the fabrication to avoid any potential electrical and thermoelectric properties´ deterioration [48]. Regarding this issue, a higher quantity of SWCNTs and consequently SDBS produced more mechanically defective nanocomposites (Figure 5).

**Figure 6:**
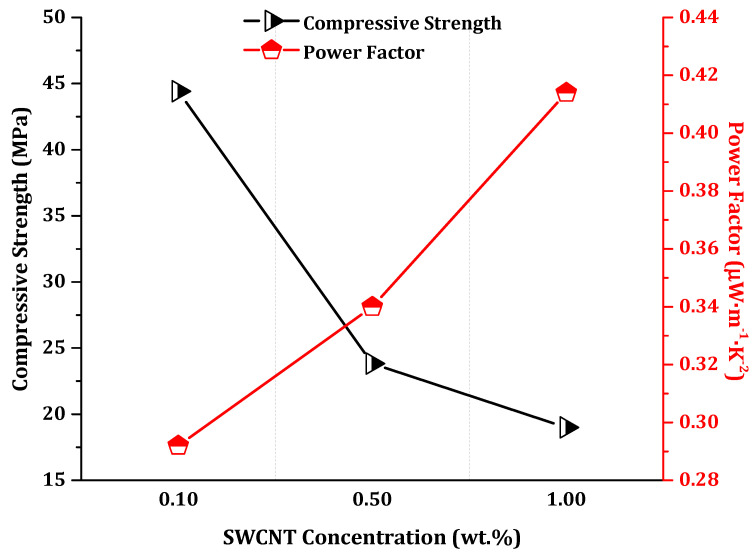
Inverse correlation of mechanical and thermoelectric properties of SWCNT-alkali-activated nanocomposites.

Overall, incorporation of CNTs into alkali-activated materials can be simultaneously beneficial and detrimental, depending on the concentration of CNTs, the applied surfactant, and the target (Figure 6). Mechanical and microstructural improvements can be achieved within low CNT-content nanocomposites, i.e., c ≤ 0.1 wt.% [13]. At higher CNT concentrations, a gradual or abrupt decline will be inevitable as can be seen here within the results (Figure 5). Nonetheless, integration of higher concentrations of CNT is very advantageous for non-conventional applications such as stimuli-responsiveness and thermoelectricity, where reinforcement is not the main objective. Therefore, in the present research, the higher concentration of SWCNTs is preferred, despite the resultant microstructural degradations (Figure 6). To achieve a higher electrical conductivity and corresponding power factor, a substantial concentration of SWCNTs, i.e., c ≫ 0.1 wt.%, is required to firmly establish a SWCNT conductive network. In this way, the significant electrical conductivity of nanocomposites could even compensate the negative declining trend of the Seebeck effect (Figure 2).

### 3.5. Microstructure of 1.0 wt.% SWCNT Nanocomposites

**Figure 7:**
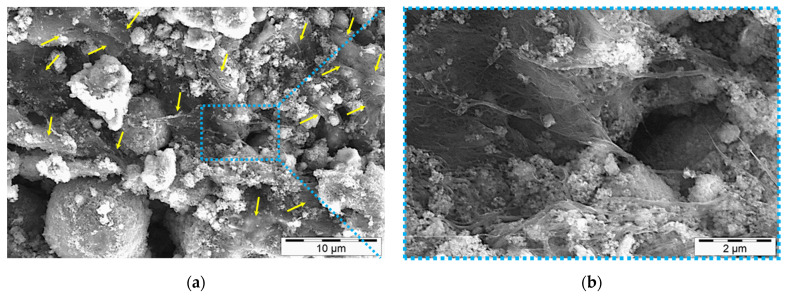
SWCNTs’ electrically conductive network generated in 3-day one-part SWCNT-alkali-activated nanocomposites, SWCNT conc. 1.0 wt.%. SWCNT agglomerates constituting the network are marked with yellow arrows. (**b**) is the higher magnification of the selected zone in (**a**), shown by a dot blue box.

The fractal conductive network of SWCNTs generated in the investigated alkali-activated matrix is illustrated in Figure 7 for 3 days of specimens. At this age, the gel structure was still under development and SWCNTs were not embedded and confined in-between, hence, better discoverable. This matrix shows an exceptional planar distribution of SWCNTs with fully established connections and links required for those observed high conductivities in Figure 2 and Table 3. It can be seen that SWCNTs’ network was spontaneously allocated throughout the developing gel of alkali-activated matrix (yellow arrows in Figure 7a). The density of the generated network (in agglomerated forms) can be better observed in the higher magnification micrograph in Figure 7b. 

**Figure 8:**
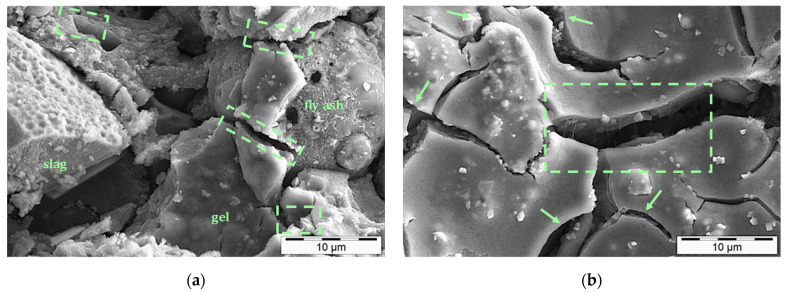
SWCNTs’ distribution in 28-day one-part SWCNT-alkali-activated nanocomposites, SWCNT conc. 1.0 wt.%. SWCNTs are highlighted with green arrows and boxes. (**a**) and (**b**) show different regions in the microstructure.

After 28 days of reaction, some fly ash and slag particles are still visible in some areas (Figure 8a). Nonetheless, in the other regions, the gel is mostly formed (Figure 8b) and spatial distribution of SWCNTs can be seen within ruptured gel zones, which are highlighted with green arrows and boxes in Figure 8a,b. In an overview, the texture looks imperfectly homogeneous and many microcracks can be found in the microstructure, representing the inferior mechanical performance of 1.0 wt.% SWCNT nanocomposites (Figure 5). However, even in these conditions, SWCNTs yet demonstrate their excellent load-bearing capacities. 

**Figure 9:**
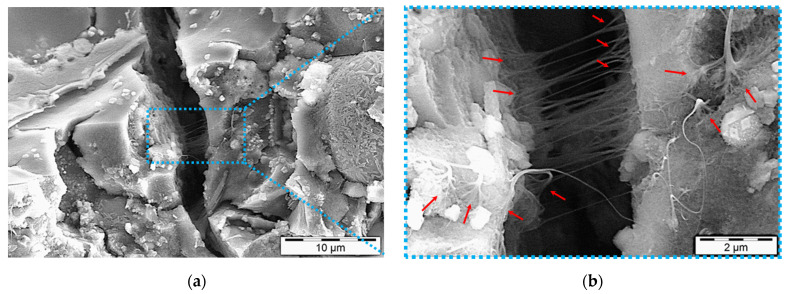
Multifunctionality of 28-day one-part SWCNT-alkali-activated nanocomposites: Integrated SWCNT electrical conductivity and mechanical reinforcement, SWCNT conc. 1.0 wt.%. Manifold configurations of SWCNTs are shown with red arrows. (**b**) is the higher magnification of the selected zone in (**a**), shown by a dot blue box.

The multifunctional capabilities of SWCNTs are demonstrated in Figure 9. Microcrack anchoring and bridging of SWCNTs (also visible in Figure 8) resulted in the matrix reinforcement while maintaining the generated tight conductive network (Figure 9a). SWCNTs around microcracks had a manifold configuration because of stress concentrations in those areas (red arrows in Figure 9b). This end-branching of agglomerates can be beneficial for electrical networking and consequently the assessed thermoelectric performance of the alkali-activated nanocomposites. 

## 4. Conclusions

SWCNT-alkali-activated nanocomposites were studied regarding their thermoelectric, mechanical, and microstructure characteristics. The evaluated thermoelectric properties demonstrated extraordinary high electrically conductive nanocomposites with a typical p-type behavior, capable of harvesting energy when exposed to a temperature gradient. The SWCNT-alkali-activated thermoelectric generator fabricated in this work additionally paves the way to potentially large-scale waste-heat capture and conversion to electricity. 

Altogether, the largest electrical conductivity and power factors were measured and calculated for 1.0 wt.% SWCNT incorporations. On the other hand, the largest Seebeck effect and mechanical reinforcement were attributed to the 0.1 wt.% SWCNT incorporations. Notwithstanding this contradiction, the multifunctionality of the nanocomposites was perfectly proven within the highest SWCNT inclusions, i.e., 1.0 wt.%. 

Further studies are needed to improve the microstructure properties of the investigated nanocomposites in a way that the high load of SWCNTs can be more effectively integrated with the preservation of the mechanical properties. Utilizing an alternative non-foaming surfactant could be a practical approach. Moreover, further research has to be conducted to maximize the power factor and the generated maximum power for future full-scale industrial applications in structure engineering with a higher amount of p-type or alternating p-n serially interconnected thermoelements. The addition of another nanomaterial or the functionalization of the CNTs could be a practical pathway to improve the Seebeck coefficient of the nanocomposites [49].

## Figures and Tables

**Table 1:** Single-walled carbon nanotube (SWCNT) specification and structure parameters (provided by OCSIAL).

CNT Content	G/D	Outer Mean Diameter	Length	Aspect Ratio	Specific Surface Area	Metal Impurities
wt.%	-	nm	µm	-	m^2^·g^−1^	wt.%
≥80	>90	1.6 ± 0.4	>5	3000	1000	≤15

**Table 2:** One-part SWCNT-alkali-activated nanocomposites’ chemical laboratory-scale mix design.

SWCNT	SWCNT	SDBS 10%	W/S	DI-Water	Fly Ash	GGBS	Sodium Disilicate
**wt.%**	**mg**	**g**	**-**	**g**	**g**	**g**	**g**
0.1	34	0.34	0.45	16.50	24	10	6
0.5	170	1.70	0.45	16.50	24	10	6
1.0	340	3.40	0.45	16.50	24	10	6

**Table 3:** Thermoelectric properties of 28-day one-part SWCNT-alkali-activated nanocomposites.

SWCNT Concentration	Electrical Conductivity	Seebeck Coefficient	Power Factor
SWCNT	*σ*	*S* (−Δ*V*/Δ*T*)	*PF* (*σ*·*S*^2^)
wt.%	S·m^−1^	μV·K^−1^	μW·m^−1^·K^−2^
0.1	9.42 × 10^2^ ± 8.9 × 10^−1^	17.6 ± 0.8	0.292 ± 0.05
0.5	1.23 × 10^3^ ± 5.1 × 10^0^	16.6 ± 0.8	0.340 ± 0.04
1.0	1.66 × 10^3^ ± 5.1 × 10^0^	15.8 ± 0.8	0.414 ± 0.04

**Table 4:** Maximum electrical power output, power density, and thermoelectric power voltage of the SWCNT-alkali-activated TEG.

Δ*T* (K)	*P_density_* (nW m^−2^)	*P_max_* (μW)	*V_TEP_* (mV)
15	23	0.085	2.1
30	171	0.251	3.8
60	372	0.695	7.2

## Data Availability

Not applicable.
